# Cell-Based Neuroprotection of Retinal Ganglion Cells in Animal Models of Optic Neuropathies

**DOI:** 10.3390/biology10111181

**Published:** 2021-11-15

**Authors:** Yue Hu, Lynn Michelle Grodzki, Susanne Bartsch, Udo Bartsch

**Affiliations:** Department of Ophthalmology, Experimental Ophthalmology, University Medical Center Hamburg-Eppendorf, 20246 Hamburg, Germany; mia.huy@outlook.com (Y.H.); l.grodzki@uke.de (L.M.G.); sbartsch@uke.de (S.B.)

**Keywords:** cell transplantation, glaucoma, neurotrophic factors, retinal ganglion cells, stem cells

## Abstract

**Simple Summary:**

Progressive loss of retinal ganglion cells in glaucoma and other optic neuropathies results in visual deterioration and eventual blindness. Treatment options for these conditions are limited. In this review, we summarize preclinical work that has demonstrated significant attenuation of ganglion cell loss after intraocular transplantations of a variety of cell types. We also discuss studies aimed at improving the outcome of cell-based neuroprotective approaches by using cells with a forced expression of neurotrophic factors or by employing combinatorial cell-based neuroprotective strategies.

**Abstract:**

Retinal ganglion cells (RGCs) comprise a heterogenous group of projection neurons that transmit visual information from the retina to the brain. Progressive degeneration of these cells, as it occurs in inflammatory, ischemic, traumatic or glaucomatous optic neuropathies, results in visual deterioration and is among the leading causes of irreversible blindness. Treatment options for these diseases are limited. Neuroprotective approaches aim to slow down and eventually halt the loss of ganglion cells in these disorders. In this review, we have summarized preclinical studies that have evaluated the efficacy of cell-based neuroprotective treatment strategies to rescue retinal ganglion cells from cell death. Intraocular transplantations of diverse genetically nonmodified cell types or cells engineered to overexpress neurotrophic factors have been demonstrated to result in significant attenuation of ganglion cell loss in animal models of different optic neuropathies. Cell-based combinatorial neuroprotective approaches represent a potential strategy to further increase the survival rates of retinal ganglion cells. However, data about the long-term impact of the different cell-based treatment strategies on retinal ganglion cell survival and detailed analyses of potential adverse effects of a sustained intraocular delivery of neurotrophic factors on retina structure and function are limited, making it difficult to assess their therapeutic potential.

## 1. Introduction

Retinal ganglion cells (RGCs) comprise a heterogeneous group of projection neurons that transmit visual information to the brain. Until now, RGCs have been classified into more than 40 subtypes according to anatomical, physiological and molecular criteria. RGC subtypes also show a striking heterogeneity in their ability to survive under different pathological conditions and to regrow their axons after injury [1,2,3,4,5,6,7,8]. Progressive loss of RGCs in neurodegenerative retinal disorders, such as ischemic, inflammatory, traumatic or glaucomatous optic neuropathies, is among the leading causes of visual deterioration and blindness. Treatment options for these conditions are either not available or of limited efficacy. For instance, an elevated intraocular pressure (IOP) is considered as the major risk factor for developing glaucoma, the most prevalent optic neuropathy. While lowering the IOP with anti-glaucomatous medications or surgical interventions is currently the only clinically proven treatment of glaucoma, the disease progresses in a significant fraction of patients despite successful management of the IOP [9,10,11,12,13]. In addition to an elevated IOP, a number of other risk factors have been implicated in the pathogenesis of glaucoma, including neurotrophic factor (NTF) deprivation, excitotoxicity, oxidative stress, neuroinflammation, vascular dysfunction, mitochondrial dysfunction or impaired neuron-glia interactions [14,15,16,17]. There is thus an urgent need to develop effective treatment options for optic neuropathies, including novel IOP-independent treatment strategies for glaucoma.

A variety of genetic or acutely induced animal models of optic neuropathies recapitulate important aspects of the diseases and are thus important means for preclinical studies aimed at developing treatment options. These include, for instance, ocular hypertension (OHT), excitotoxicity or ischemia/reperfusion models [18,19,20,21,22,23,24]. However, most preclinical studies have used optic nerve injury models to evaluate the impact of treatment strategies on RGC survival. Degeneration of RGCs in these models is usually induced by an intraorbital crush or transection of the optic nerve, resulting in a rapid and extensive loss of RGCs, with little variability between animals, greatly facilitating the analysis of treatment efficacies.

Diverse treatment strategies are being tested for their efficacy tin rescuing RGCs from cell death, such as dietary modifications, electrical stimulation therapy, immunomodulatory strategies or gene therapy [25,26,27,28,29,30,31,32]. At late stages of the disease, the only treatment option is a functional replacement of the lost RGCs. This aim might be achieved by the transplantation of RGCs derived from embryonic stem cells or induced pluripotent stem cells. While the generation of RGCs from pluripotent stem cells has made considerable progress, RGC replacement by cell transplantation is faced with a number of problems that need to be solved before restoration of meaningful vision might be achieved, such as functional integration of specific RGC subtypes into the dystrophic retinas or long-distance growth of axons and the formation of functional synapses in visual target regions of the brain [33,34,35,36,37]. Most current therapeutic approaches therefore aim to delay or prevent the degeneration of those RGCs that are still viable at initial stages of the disease.

Neuroprotective strategies are of potential clinical interest, as a neuroprotective treatment that effectively promotes RGC survival might be applicable to different optic neuropathies independent of the specific etiology. A large number of neuroprotective agents have been demonstrated to promote RGC survival in preclinical settings, including the non-competitive N-methyl-d-aspartate receptor antagonist memantine or the α2-adrenergic receptor agonist brimonidine. Results from clinical trials with these agents were, however, either inconclusive or showed no positive therapeutic effects in glaucoma patients [25,38,39,40]. Significant protection of RGCs has also been demonstrated in diverse animal models of optic neuropathies after intraocular administration of various NTFs, such as ciliary neurotrophic factor (CNTF), glial cell line-derived neurotrophic factor (GDNF), brain-derived neurotrophic factor (BDNF) and others. Because NTFs usually have a short half-life and are unable to cross the blood-retina barrier, they need to be administered locally and continuously to effectively promote RGC survival. This aim might be achieved through a viral or nonviral gene transfer to the dystrophic retinas or through intraocular implantations of NTF-loaded slow-release devices [14,25,41,42,43,44]. Intraocular transplantations of diverse genetically non-modified cell types have also been shown to confer significant protection to RGCs under a variety of pathological conditions through secretion of immunomodulatory mediators, NTFs and exosomes [34,45,46,47,48,49,50].

In the following, we first summarize preclinical work that has evaluated the impact of intraocular transplantations of diverse genetically non-modified cell types on RGC survival. We then summarize studies that have performed intraocular transplantations of genetically modified cells with a forced expression of NTFs to augment RGC survival. Finally, we discuss combinatorial cell-based neuroprotective approaches aimed at increasing the rate and durability of RGC survival achieved with cell-based monotherapies.

## 2. Diverse Genetically Non-Modified Cell Types Promote Retinal Ganglion Cell Survival

Significant attenuation of RGC loss in animal models of optic neuropathies has been observed after intraocular transplantations of a variety of genetically non-modified cell types, such as mesenchymal stem cells (MSCs), olfactory ensheathing cells (OECs), oligodendrocyte progenitor cells (OPCs), Schwann cells (SCs) and others [43,45,47,48,51,52,53,54,55,56].

### 2.1. Mesenchymal Stem Cells

MSCs comprise a population of multipotent adult stem cells that can be relatively easily isolated from different tissues with low invasiveness and subsequently expanded in vitro, permitting their use in autologous transplantation settings and thus avoiding rejection problems associated with most other stem cell types [57,58]. Due to their anti-apoptotic and immunomodulatory activities, MSCs are considered as promising candidate cells for the development of therapies for the treatment of neurodegenerative diseases [45,46,59,60]. MSCs can be derived, for instance, from bone marrow (bone marrow-derived MSCs (BMSCs)), adipose tissue (adipose-derived MSCs, (ADSCs)), dental tissues, such as dental pulp (dental pulp stem cells, (DPSCs)), umbilical cord blood (umbilical cord blood-derived MSCs (UCB-MSCs)) or umbilical cord Wharton’s jelly (umbilical cord Wharton’s jelly-derived MSCs (WJ-MSCs)) [45,51,58]. Because there is no unique marker, minimal defining criteria have been suggested for MSCs, including (i) adherence to cell culture plastic under standard culture conditions, (ii) expression of cell surface markers, such as CD73, CD90 and CD105, (iii) absence of cell surface markers, such as CD14, CD34, CD45 and HLA-DR, and (iv) the ability to differentiate into adipogenic, chondrogenic and osteogenic lineages [57,61].

#### 2.1.1. Bone Marrow-Derived Mesenchymal Stem Cells

Intravitreal transplantations of BMSCs into rat OHT models induced by photocoagulation of the trabecular meshwork [62,63], ligation or cauterization of episcleral veins [64,65], or intracameral injections of transforming growth factor-β1 (TGF-β1) [66] or hyaluronic acid [67] have been shown to rescue a significant fraction of RGCs from cell death. Transplantations of BMSCs either one week before [62] or at the time of OHT induction [66], for instance, significantly attenuated the loss of axons in the optic nerve [62] or of brain-specific homeobox/POU domain protein-3A (BRN3A)-positive RGC somata [66] over a period of about 4 or 5 weeks. The latter study additionally reported preservation of the retinal nerve fiber layer and retina function in treated animals, as assessed by optical coherence tomography (OCT) and electroretinogram (ERG) recordings, respectively. A significant rescue of RGCs in OHT models was also observed when BMSCs were grafted some time after induction of an elevated IOP. For instance, when BMSCs were grafted 1 week after OHT induction and animals were analysed 4 weeks later, the number of surviving RGCs was ~1.3-fold higher than in vehicle-injected controls. Treated retinas contained more cells expressing anti-inflammatory cytokines, such as interleukin-1 receptor antagonist (IL-1RA), and fewer cells expressing pro-inflammatory cytokines, such as interferon-γ (IFN-γ) and tumor necrosis factor-α (TNF-α) [67]. Furthermore, it was shown that rats with grafted BMSCs bilaterally injected into the vitreous cavity 6 weeks after IOP elevation and analysed 4 weeks later performed better in a visual behavioral test than controls. Improvement of retina function correlated with the presence of more viable RGCs in eyes with grafted BMSCs than in control eyes, as assessed by retrograde axonal tracings from the superior colliculus [63]. Finally, Yu and colleagues performed intravitreal BMSC injections 8 weeks after cauterization of the episcleral veins of rats and analysed the retinas 4 weeks later [64]. The higher numbers of viable RGCs in treated eyes, as compared to vehicle-injected glaucomatous eyes, coincided with significantly elevated expression levels of fibroblast growth factor-2 (FGF-2) and CNTF, while levels of GDNF and BDNF were not significantly different from those in control retinas [64]. Another study found that a significant protection of RGCs in a rat OHT model correlated with a rapid and significant decrease in the IOP after a single injection of BMSCs into the anterior chamber. Based on in vitro experiments, authors suggested that BMSCs support RGC survival in hypertensive eyes through the preservation of trabecular meshwork integrity [65].

The ability of BMSCs to promote RGC survival and/or axon regeneration was also evaluated in optic nerve injury models [68,69,70,71]. One study used specific culture conditions to elevate the expression of BDNF and GDNF in human BMSCs. Intravitreal injections of these cells into a rat optic nerve transection (ONT) model resulted in the presence of significantly more viable RGCs than in vehicle-injected eyes. It should be noted, however, that retinas were analysed as early as 8 days after the nerve lesion. It is worth noting that rat BMSCs used in the same study and stimulated to express elevated levels of BDNF (but not GDNF) did not rescue RGCs from lesion-induced cell death [68]. In striking contrast, rat BMSCs conferred robust protection to RGCs in a rat optic nerve crush (ONC) model over a period of at least 4 weeks [70]. The grafted cells additionally stimulated axon regrowth into the distal optic nerve stumps [70], in line with other studies [71,72]. Immunohistochemistry revealed elevated expression levels of FGF-2 and the pro-inflammatory cytokine interleukin-1β (IL-1β) in the ganglion cell layer of cell-treated retinas, as compared to saline-treated retinas [70]. Murine BMSCs also promoted the survival of injured RGCs, as demonstrated in a mouse ONC model. The study found markedly elevated levels of BDNF in treated retinas and presented experimental evidence for a critical role of BDNF and GDNF in mediating the rescue effect [69] (see Section 2.3).

#### 2.1.2. Umbilical Cord-Derived Mesenchymal Stem Cells

MSCs derived from umbilical cords, which can be noninvasively harvested and are devoid of ethical concerns, such as umbilical cord blood-derived MSCs (UCB-MSCs) and umbilical cord Wharton’s jelly-derived MSCs (WJ-MSCs) have also been tested for their ability to promote RGC survival. Intravitreally transplanted WJ-MSCs, for instance, have been reported to promote the survival of axotomized RGCs in a rat ONC model [73]. However, the neuroprotective effect was short-term and only evident 14 days but not 30 days after the lesion. The transient survival-promoting effect was attributed to a progressive loss of donor cells and a concomitant decrease in the levels of neurotrophic factors (i.e., nerve growth factor (NGF), BDNF, vascular endothelial growth factor (VEGF) and CNTF) and immunomodulators (i.e., TGF-ß1 and prostaglandin E_2_ (PGE_2_)). Noteworthily, the transplanted cells adversely affected retina structure, caused retinal detachments and the formation of retinal folds and stimulated a massive infiltration of microglia/macrophages [73]. In striking difference to the short-term effects observed by Millan-Rivero and colleagues, another study reported a more sustained RGC rescue using the same cell type and the same animal model [74]. A total of 60 and 120 days after the treatment, the number of β-III-tubulin-positive RGCs was ~2-fold higher in treated eyes, as compared to vehicle-injected eyes, accounting for ~23% and ~10% of the normal RGC population, respectively. The treatment preferentially protected large-sized RGCs, presumably including αRGCs, an RGC subtype known to be particularly resistant to injury [4]. The grafted cells additionally stimulated long-distance axon regeneration into the distal optic nerve stumps [74]. WJ-MSCs also protected RGCs in a rat OHT model induced by intracameral injections of polystyrene microbeads as assessed by RGC counts in retina flatmounts and axon counts in the optic nerve. However, the neuroprotective effect was minor, albeit analyses were performed as early as 2 weeks after the treatment [75]. Immunoblot analysis and immunohistochemistry revealed increased levels of BDNF and GDNF and decreased levels of glial fibrillary acidic protein (GFAP) in WJ-MSC-treated retinas, as compared to vehicle-injected retinas [75].

Intravitreal injections of MSCs isolated from umbilical cord blood transiently improved RGC survival to some extent for about 3 weeks after optic nerve injury [76]. Expression of growth-associated protein 43 (GAP43) in treated retinas was increased for a similar period of time after the lesion, but axon regeneration in the optic nerve was not analysed [76]. MSCs cultured as spheroids have been shown to display more pronounced anti-inflammatory and anti-apoptotic activities than adherently cultivated MSCs, suggesting that they might have a greater therapeutic potential [77,78]. Huang and colleagues therefore compared the impact of UCB-MSCs cultured either as spheroids (3D-UCB-MSCs) or monolayers (2D-UCB-MSCs) on RGC survival, axon regeneration and retina function in a mouse ONC model [79]. Analyses revealed that 2D-UCB-MSCs rescued a significant fraction of RGCs from lesion-induced cell death during the first two weeks after the lesion, while numbers of viable RGCs in animals treated with 3D-UCB-MSCs were similar to those in saline-treated eyes. Similarly, significant axon regeneration into the distal nerve stumps was only observed after transplantations of UCB-MSCs cultured as monolayer but not after transplantations of UCB-MSCs cultured as spheroids. Finally, amplitudes of flash visual evoked potentials were preserved in the 2D-UCB-MSC group but not in the 3D-UCB-MSC group.

#### 2.1.3. Adipose-Derived Mesenchymal Stem Cells

Emre and colleagues compared the efficacy of adipose-derived mesenchymal stem cells (ADSCs) and BMSCs (see Section 2.1.1) to rescue RGCs in hypertensive eyes and found that both cell populations promoted RGC survival to a similar extent and over a similar period of time. The overall changes in expression levels of pro- and anti-inflammatory cytokines were also similar in BMSC- and ADSC-treated retinas [67]. In striking difference to these findings, another study found no beneficial effects of ADSCs in a rat open-angle glaucoma model induced by intracameral injections of TGF-β1. Analyses of animals 5 weeks after OHT induction and cell transplantation revealed that ADSCs neither prevented RGC death or thinning of the retinal nerve fiber layer nor preserved RGC function, as indicated by the positive scotopic threshold responses in ERG recordings [66]. Of note, dental pulp stem cells (see Section 2.1.4) and, to a lesser extent, BMSCs promoted RGC survival and partially preserved RGC function in the same glaucoma model [66]. Observations indicate that MSCs isolated from different tissues differ in their ability to attenuate glaucomatous-like pathologies, with ADSCs being the least effective cell type (see Section 2.1.4). In general agreement with this notion, ADSCs protected RGCs in a rat ONC model to only a minor extent and for only a limited period of time [80].

#### 2.1.4. Dental Pulp Stem Cells

First evidence that MSC-like cells isolated from dental pulp (dental pulp stem cells (DPSCs)) show a greater potential to promote RGC survival and axon regeneration than MSCs isolated from other tissues was provided in a rat ONC model [72]. Using a transwell co-culture system, Mead and colleagues first showed that rat DPSCs were significantly more effective in promoting survival and neurite outgrowth from β-III-tubulin-positive primary retinal cells than BMSCs. The more pronounced neuroprotective effects conferred by DPSCs, as compared to BMSCs, correlated with significantly higher secretion levels of NGF and BDNF. Importantly, authors additionally presented experimental evidence that part of the survival- and neurite outgrowth-promoting activities of DPSCs and BMSCs were mediated through neurotrophins. In line with the in vitro data, the study then showed that DPSCs were also more effective than BMSCs in protecting RGCs and in stimulating regrowth of injured axons in vivo in an optic nerve injury model [72]. A follow-up in vitro study compared the neuroprotective and neurite growth-promoting activities of human DPSCs, BMSCs and ADSCs and confirmed that, overall, DPSCs were the most effective cell type, followed by BMSCs and, finally, ADSCs. While all three cell populations secreted a variety of neuroprotective factors, including NGF, GDNF, VEGF and platelet-derived growth factor (PDGF), they differed markedly in expression levels of specific factors. For instance, similar to rat DPSCs, human DPSCs expressed particularly high levels of NGF and BDNF. ADSCs, on the contrary, expressed only low levels of GDNF and neither BDNF nor neurotrophin-3 (NT-3) [81]. Differences in the NTF secretome thus explain, at least in part, the different efficacies of DPSCs, BMSCs and ADSCs in attenuating the morphological and functional phenotype of the rat open-angle glaucoma model discussed above (see Section 2.1.3) [66].

### 2.2. Other Cell Types

Olfactory ensheathing cells (OECs) support the growth of axons from the constantly replaced sensory neurons in the olfactory epithelium. These specialized glial cells express a multitude of NTFs, cell recognition molecules and other proteins implicated in axon growth and are therefore considered as promising candidates for cell-based therapies in the nervous system [48,82,83]. In the visual system, intravitreally grafted OECs immigrated into the optic nerve head of hypertensive rat eyes and attenuated the loss of RGC axons over a period of at least 4 weeks [84]. OECs also protected RGCs against lesion-induced cell death when grafted into the distal stump of transected optic nerves. However, the rescue effect was short-term and only detectable 7 but not 14 days after the lesion. Improved RGC survival coincided with a transient increase in BDNF levels, while levels of GDNF, leukemia inhibitory factor (LIF), NGF and NT-3 were similar to those in control eyes [85]. Finally, OECs placed at the lesion site of transected rat optic nerves immigrated over considerable distances into the distal nerve stumps and supported long-distance axon regeneration. Whether the treatment also promoted RGC survival was, however, not analysed [86].

Using a rat OHT model, Bull and colleagues identified oligodendrocyte progenitor cells (OPCs) as another cell type conferring neuroprotection to RGCs. However, improved RGC survival was only observed when OPC transplantations were combined with zymosan-induced inflammation. Authors suggested that OPCs promoted RGC survival through the release of NTFs upon activation by inflammatory cells [87].

For a detailed discussion of the extensive literature about the neuroprotective and axon regeneration-promoting potential of Schwann cells and peripheral nerve grafts, the reader is referred to excellent reviews covering this topic [43,88,89,90].

### 2.3. Proposed Mechanisms of Neuroprotection Conferred by Grafted Cells

While it is well established that a variety of cell types promote RGC survival and stimulate axon regeneration, precise knowledge about how these effects are mediated is limited. Several lines of evidence indicate a critical role of secreted factors. In vitro experiments, for instance, demonstrated improved survival when RGCs were cultivated in conditioned medium from MSCs or OECs [91,92] or maintained in transwell co-cultures together with DPSCs, BMSCs or ADSCs [81,93,94,95,96]. In vivo, significant protection of RGCs was conferred by grafted cells that remained in the vitreous cavity [64,66,70,97,98]. Furthermore, intraocular injections of conditioned medium from BMSC cultures attenuated the loss of cells in the ganglion cell layer of ischemic retinas [99,100].

As detailed above, most studies have attributed the survival-promoting effects to the secretion of various NTFs acting either directly or indirectly via stimulation of other retinal cell types on RGCs. In line with this view, mesenchymal stem cells, olfactory ensheathing cells, oligodendrocyte lineage cells and Schwann cells have all been shown to express a multitude of NTFs implicated in RGC protection, such as BDNF, GDNF, CNTF, NT-3, FGF-2, LIF, PDGF, VEGF, insulin-like growth factor-1 (IGF-1) and others [64,68,81,85,89,91,97,101,102,103,104,105,106]. Vice versa, analyses of the secretome of these cells have led to the identification of novel neuroprotective factors for RGCs, such as osteonectin expressed by Schwann cells [107] or VGF nerve growth factor inducible (VGF) expressed by DPSCs [81]. While these findings suggest a critical role of the NTF secretome in promoting RGC survival, only a few studies have provided direct experimental evidence in support of this view. BDNF and VEGF, for instance, were identified as important NTFs in conditioned media from OECs and BMSCs, respectively, as indicated by markedly reduced RGC survival rates in the presence of function-blocking antibodies [92,106]. Another study showed that inhibition of PDGF signalling attenuated the neuroprotective effects of human MSCs on RGCs in co-cultured rat (but not human) retina explants [91,108]. Using inhibitors for various NTF receptors, NGF, BDNF, NT-3, GDNF, VEGF, PDGF-AA and PDGF-AB/BB were shown to contribute to the survival-promoting activity of DPSCs on co-cultured RGCs, with some of these factors also mediating part of the neuroprotective effects of BMSCs and ADSCs [81]. Finally, in vivo experiments demonstrated that a small interfering RNA (siRNA)-mediated knockdown of BDNF or GDNF in BMSCs abolished the survival-promoting effects of these cells in a mouse ONC model [69].

Exosomes are secreted nanosized vesicles of endosomal origin through which proteins, lipids, messenger RNAs (mRNAs) and microRNAs (miRNAs) are delivered to recipient cells [109]. Recent studies have shown that MSC-derived exosomes promote RGC survival in vitro and in vivo in optic nerve injury [110,111,112,113,114] and glaucoma models [115,116]. While the mechanisms through which these effects are mediated are largely unknown, improved RGC survival has been mainly attributed to miRNA-mediated modulation of gene expression in RGCs rather than to exosomal proteins [111,116].

## 3. Retinal Ganglion Cell Protection Using Genetically Modified Cells

A variety of NTFs have been shown to attenuate the loss of RGCs under various pathological conditions. However, because of short half-life times and the inability to cross the blood-retina barrier, significant long-term protection was only observed when these proteins were administered locally and continuously [14,43,44]. While a virus-mediated gene transfer of NTFs is the most frequently used delivery strategy, other strategies have also been successfully employed, such as a non-viral gene transfer or intraocular implantations of NTF-loaded slow-release devices [14,32,42,44,117,118]. Intraocular transplantations of cells genetically modified to overexpress NTFs represent another strategy to continuously administer NTFs to dystrophic retinas (Table 1). Cell-based approaches provide the possibility of determining and adjusting the amount of the administered NTF in vitro prior to cell transplantation. Furthermore, the intrinsic neuroprotective activity of certain cell types, in combination with the neuroprotective activity of the overexpressed NTF, might result in enhanced RGC survival rates. Finally, cell-based neuroprotective strategies can be translated into clinical applications, as discussed in more detail below.

### 3.1. Brain-Derived Neurotrophic Factor

Brain-derived neurotrophic factor (BDNF) is a member of the neurotrophin family of growth factors and signals through two different types of transmembrane receptors, the neurotrophin receptor p75 and the high-affinity receptor tropomyosin receptor kinase B (TrkB) [131,132,133]. Administration of the neurotrophin to dystrophic retinas has been demonstrated to potently promote RGC survival in animal models of various optic neuropathies [134,135,136,137,138]. However, the survival-promoting effect of BDNF was only transient, due, at least in part, to a limited availability of TrkB on RGCs [139]. The efficacy of a cell-based administration of BDNF to rescue RGCs from cell death was tested in vitro using retrovirally modified rat astrocytes [119]. When dissociated retinas from rat embryos were maintained for 3 days in the presence of medium conditioned by the BDNF-overexpressing astrocytes, the number of RGCs was markedly increased by a factor of 15 when compared to cultures maintained in control medium [119]. Similarly, MSCs transduced with a BDNF-encoding lentiviral vector promoted the survival of co-cultured RGC-5 cells treated with either glutamate or H_2_O_2_ more effectively than control MSCs [120]. To evaluate the efficacy of BDNF-overexpressing MSCs in promoting RGC survival in vivo, cells were intravitreally grafted into a rat OHT model 2 days after induction of an elevated IOP [121]. Eyes treated with BDNF-overexpressing MSCs contained significantly more RGCs than eyes treated with MSCs expressing a reporter gene only over a time period of at least 40 days after cell transplantation. Furthermore, there was a better preservation of retina function, as assessed by ERG recordings and analyses of the pupillary light reflex [121], demonstrating that the forced expression of the neurotrophin enhanced the beneficial impact of MSCs on retina structure and function. Improved survival of RGCs was also observed in a rat ONC model after intravitreal or subretinal injections of BDNF-overexpressing neural progenitor cells [122].

### 3.2. Ciliary Neurotrophic Factor

Ciliary neurotrophic factor (CNTF), a member of the interleukin-6 family of cytokines [140], is probably the most extensively studied NTF in the context of neurodegenerative retinal disorders. Similar to BDNF, the cytokine has been shown to attenuate RGC loss in animal models of various optic neuropathies. CNTF additionally stimulates long-distance regeneration of axotomized RGC axons into the distal optic nerve stumps [137,141,142,143,144,145,146,147,148].

**Figure 1:**
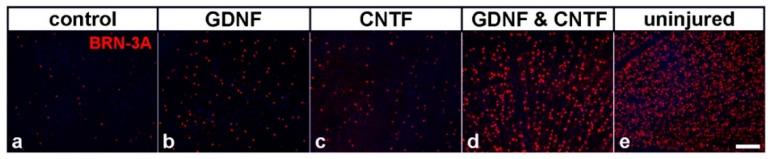
Intravitreally co-grafted GDNF- and CNTF-overexpressing neural stem cells synergistically promote the survival of RGCs in a mouse optic nerve crush model over an extended period of time. RGCs were stained with anti-BRN3A antibodies in retinal flatmounts eight months after an intraorbital optic nerve crush and intravitreal cell transplantation (**a**–**d**). The density of RGCs in eyes with grafted GDNF- (**b**) or CNTF- (**c**) overexpressing cells was similar and significantly higher than in eyes with grafted control cells that expressed a fluorescent reporter protein only (**a**). Note the pronounced rescue of axotomized ganglion cells in animals that had received co-transplantations of the GDNF- and CNTF-overexpressing cell lines (**d**). A retinal flatmount from a healthy, uninjured animal is shown for comparison (**e**). Bar: 50 µm. Figure adapted from Dulz et al. (2020).

**Figure 2:**
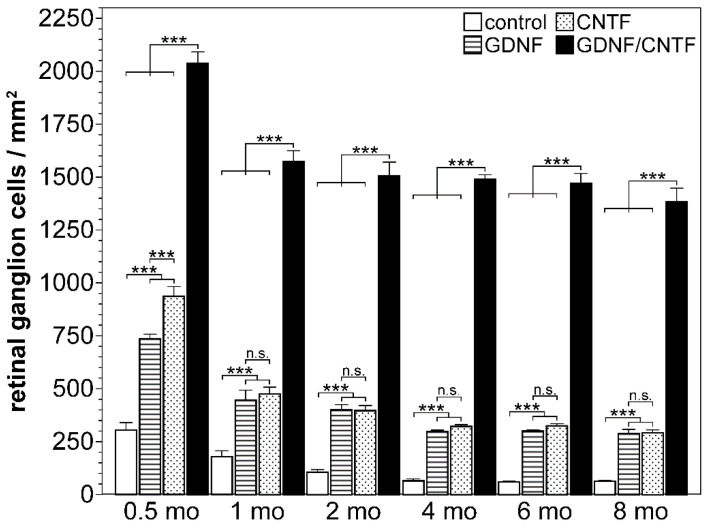
Simultaneous cell-based administration of GDNF and CNTF confers pronounced and long-lasting protection against axotomy-induced cell death. The density of BRN-3A-positive ganglion cells in retinal flatmounts at different time points after an intraorbital optic nerve crush and intravitreal transplantation of control cells (open bars), GDNF- (striped bars), CNTF- (dotted bars), or GDNF- and CNTF- (filled bars) overexpressing neural stem cell lines. The number of viable ganglion cells in GDNF- or CNTF-treated retinas was significantly higher than in control retinas at all post-lesion time points. Note the pronounced rescue of axotomized ganglion cells in retinas simultaneously treated with GDNF and CNTF when compared to retinas treated with either factor alone. Each bar represents the mean (±S.E.M.) of six animals. n.s.: not significant, ***: *p* < 0.001; two-way ANOVA followed by a Bonferroni post hoc test. Figure adapted from Dulz et al. (2020).

Peripheral nerve sheaths repopulated ex vivo with adult Schwann cells and grafted onto the lesioned optic nerves of rats stimulate axon regeneration [149]. To analyse whether a forced expression of CNTF improves the effects of Schwann cells on RGC survival and axon regeneration, nerve sheaths were repopulated with Schwann cells lentivirally modified to overexpress the cytokine. Four weeks after transplantation of the CNTF-producing grafts, both RGC survival and axon regeneration were significantly increased when compared to sheaths that were repopulated with control Schwann cells modified to express the reporter protein green fluorescent protein (GFP) [123]. Overexpression of CNTF thus further enhanced the intrinsic survival- and regeneration-promoting properties of Schwann cells, similar to the observations with BDNF-overexpressing MSCs. To evaluate the long-term impact of an intravitreal cell-based delivery of CNTF in an optic nerve injury model, our group has used a lentiviral vector to stably express the cytokine in adherently cultivated neural stem cells (NSCs) [150,151]. Cells maintained under the particular culture conditions used in this study represent a homogeneous population of clonogenic self-renewing stem cells [152,153]. Intravitreal injections of a CNTF-expressing clonal NSC line into adult mice 1 day after an intraorbital crush resulted in a long-lasting, albeit minor, rescue of axotomized RGCs, with ~7% RGCs still alive 8 months after the lesion, as compared to ~2% viable RGCs in control retinas (Figure 1 and Figure 2) [126]. It is worth noting that we observed no significant decrease in the number of surviving RGCs between the second and eighth month after treatment (Figure 2) [124,125,126]. The cell-based treatment additionally stimulated long-distance regeneration of some RGC axons into the distal optic nerve stumps [124]. Transplantations of a clonally derived NSC line that expressed only a reporter gene had no effect on RGC survival or axon regrowth, as indicated by a comparison with eyes injected with the vehicle solution.

### 3.3. Glial Cell Line-Derived Neurotrophic Factor

GDNF, a member of the GDNF family of ligands [154,155], is another NTF that has been shown to effectively promote RGC survival under various pathological conditions when applied as a recombinant protein or delivered via a virus-mediated gene transfer, electroporation or GDNF-loaded microspheres [156,157,158,159,160,161,162,163]. To analyse the long-term impact of a cell-based administration of GDNF on the survival of axotomized RGCs and to compare the outcome with that obtained with the CNTF-expressing NSC clone discussed above, we generated a GDNF-expressing clonal NSC line, which protected axotomized RGCs with a similar efficacy against a lesion-induced cell death as the CNTF NSC line [125]. Long-term experiments revealed RGC survival rates in GDNF-treated retinas that were similar to those found in CNTF-treated retinas for up to 8 months after transplantation (Figure 1 and Figure 2) [126]. Noteworthily, there was no significant loss of RGCs in GDNF-treated retinas between the second and eighth month after the lesion and a low variability in RGC survival rates between animals [126], similar to the results obtained with the CNTF-expressing NSCs. The combined findings indicate that intravitreal transplantations of stably modified clonal cell lines represent a useful means to continuously and reproducibly deliver defined quantities of NTFs to dystrophic retinas over an extended period of time.

### 3.4. Other Neuroprotective Factors

Improved RGC survival was also observed in a mouse OHT model after intravitreal injections of human neural progenitor cells nucleofected with an IGF-1-encoding plasmid [127]. The modified neural progenitor cells supported survival and stimulated neurite outgrowth from co-cultured RGCs in vitro, with both effects being blocked by IGF-1-signalling inhibitors or IGF-1 receptor antagonists. More importantly, the IGF-1-expressing neural progenitor cells completely prevented the loss of RGCs in the hypertensive eyes for up to at least 30 days after cell transplantation [127]. Other studies have analysed the impact of a cell-based intraocular delivery of pigment epithelium-derived factor (PEDF) [129], FGF-2 [128] or crystallin beta B2 (CRYBB2) [130] on RGC survival in rat optic nerve injury models. Subretinally injected PEDF-overexpressing NSCs rescued more axotomized RGCs from cell death and stimulated greater axon regrowth for at least 500 µm into the distal optic nerve stumps than repeated intravitreal injections of recombinant PEDF [129]. Amniotic epithelial cells endogenously express several NTFs, such as BDNF, NT-3 and NGF, and display immunomodulatory properties [164]. When these cells were grafted to the lesion site of transected optic nerves, the number of surviving RGCs was significantly increased when compared to vehicle injections. However, RGC survival rates were significantly higher when the cells were lentivirally modified to express FGF-2 prior to the transplantation [128]. Finally, intravitreal injections of neural progenitor cells nucleofected with a plasmid encoding CRYBB2 promoted the survival of axotomized RGCs with a similar efficacy as injections of the recombinant protein [130]. The impact of a cell-based administration of PEDF, FGF-2 or CRYBB2 on RGCs survival was, however, evaluated as early as ~4 weeks after optic nerve injury and cell transplantation, similar to most other studies that have used genetically modified cells to promote RGC survival (Table 1).

## 4. Boosting Retinal Ganglion Cell Survival with Cell-Based Combinatorial Neuroprotective Approaches

To increase RGC survival rates and survival times achieved with intraocular transplantations of genetically nonmodified or modified cells, cell-based monotherapies were combined with a variety of other treatment strategies (Table 2). MSCs and Schwann cells have both been shown to rescue RGCs and to stimulate axon regeneration after injury. To analyse whether both cell types promote RGC survival in a cooperative manner, they were intravitreally co-grafted into rats with intraorbitally crushed optic nerves. The combined treatment promoted the survival of significantly more injured RGCs than separate transplantations of each cell type [165]. Another study [166] combined intravitreal transplantations of WJ-MSCs with an AAV vector-mediated gene transfer of PEDF, a member of the serine proteinase inhibitor family that has been shown to promote RGC survival in vitro and in vivo [129,167,168]. The PEDF gene therapy promoted RGC survival in a rat ONC model for at least four weeks after the lesion, but—different to other reports—did not stimulate axon regeneration. The pro-survival effect of PEDF coincided with increased levels of FGF-2, decreased levels of IL-1ß and attenuation of reactive microgliosis and astrogliosis when compared to eyes injected with a control vector. The combination of the gene therapy and the cell-based approach resulted in significantly higher numbers of viable RGCs and about twice as many regrowing axons in close vicinity to the lesion site than injections of MSCs only [166].

To enhance RGC survival rates observed after transplantations of olfactory ensheathing cells, cell-based treatments were combined with intravitreal injections of recombinant neuroprotective factors. In the first study, the optic nerve of rats was incompletely crushed, and transplantations of OECs into the optic nerves close to the site of injury were combined with intravitreal injections of recombinant GDNF [169]. Latencies and amplitudes of flash visual evoked potentials recovered faster in animals treated simultaneously with OECs and GDNF than in animals treated with either the cells or the NTF. Although quantitative analyses were not performed, the combinatorial treatment also appeared to stimulate axon regrowth, as compared to each treatment alone [169]. Another study combined OEC injections into the injury site of transected optic nerves with intravitreal injections of recombinant CNTF at the time of nerve lesioning. Again, the combinatorial treatment rescued more RGCs from lesion-induced cell death and stimulated more RGCs to regrow their axons than either treatment alone [170]. Finally, OECs and olfactory fibroblasts were transplanted into the lesion site, and α-crystallin was injected into the vitreous cavity of a rat ONC model [171]. Analyses 4 weeks after the crush revealed the presence of significantly more viable RGCs in retinas simultaneously treated with cells and α-crystallin than in retinas treated with cells only or α-crystallin only. In addition, axons in the combinatorial group extended over significantly longer distances into the distal optic nerve stumps than in the cell-treated group 1 month after the lesion but not at later post-lesion time points [171].

Zhou and colleagues combined intravitreal injections of retinal stem cells (RSCs) with an immunomodulatory approach to enhance RGC survival in a rat OHT model [172]. Animals were vaccinated with copolymer-1 (Cop-1) at the time of elevated IOP induction through photocoagulation of the episcleral veins and the limbal plexus. Intravitreal injections of syngenic RSCs were performed 7 days later. The combinatorial treatment rescued significantly more RGCs than the treatment with either COP-1 or RSCs and improved survival rates correlated with elevated levels of BDNF and IGF-1 in treated retinas [172].

## 5. Retinal Ganglion Cell Protection with Neurotrophic Factor Combinations

Increased survival rates of RGCs observed after intraocular transplantations of some nonmodified cell types are thought to be mediated, at least in part, through cooperative neuroprotective activities of multiple NTFs released from these cells (see above). Experimental evidence in support of this view was provided in an early in vitro study using purified rat RGCs [174]. RGC survival rates were increased, for instance, in cultures simultaneously treated with insulin and BDNF when compared to cultures treated with insulin or BDNF only. Similarly, more surviving RGCs were found after administration of insulin, BDNF and CNTF than after administration of insulin and BDNF, insulin and CNTF, or BDNF and CNTF [174]. Similarly, co-treatments of retinal cultures maintained in the presence of central nervous system (CNS) myelin extracts with FGF-2, NT-3 and BDNF markedly increased the number of viable RGCs and the length of RGC neurites when compared to cultures treated with each factor individually [173]. Importantly, cooperative neuroprotective activities of NTFs were also observed in optic nerve injury models in vivo. Intravitreal co-injections of recombinant BDNF and GDNF, for instance, rescued significantly more RGCs from lesion-induced cell death in a rat optic nerve transection model than injections of either BDNF or GDNF [163]. Similarly, simultaneous treatments with recombinant GDNF and BDNF, neurturin (NRTN) and BDNF, or NRTN and GDNF promoted the survival of axotomized RGCs more effectively than treatments with either factor alone [175].

### Cell-Based Administration of Neurotrophic Factor Combinations

Based on the observation that a combination of FGF-2, NT-3 and BDNF cooperatively promoted RGC survival and neurite outgrowth from RGCs in vitro, authors evaluated the impact of NTF combinations on RGC survival and axon regeneration in a rat optic nerve transection model in vivo using intravitreal injections of genetically modified fibroblasts [173]. The cell-based administration of the FGF-2/NT-3/BDNF triple combination synergistically increased the number of RGCs that had survived the lesion and had regrown their axon at least 2 mm into the distal optic nerve stump 20 days after the nerve transection when compared to the administration of FGF-2/NT-3 or FGF-2/BDNF dual combinations or the separate administration of each individual factor. However, the marked rescue effect was transient, with only a few RGCs remaining 50 days post-lesion [173].

Co-transplantations of clonally derived cell lines with a stable overexpression of NTFs might represent a means to continuously deliver defined quantities of multiple neurotrophic factors in defined ratios to dystrophic retinas. To analyse the impact of a cell-based co-administration of CNTF and GDNF on RGC survival in an optic nerve crush model, we established clonal NSC lines that stably overexpressed CNTF or GDNF and protected RGCs from lesion-induced cell death with a similar efficacy (see Section 3.2 and Section 3.3). Noteworthily, we found that intravitreal co-injections of both NSC lines resulted in a pronounced synergistic rescue of injured RGCs (Figure 1 and Figure 2). In retinas simultaneously treated with GDNF and CNTF, ~38% of all RGCs were still viable two months after the lesion and cell transplantation, ~4-fold more than in retinas treated with either factor alone and ~14-fold more than in control retinas [125]. Because optic neuropathies such as glaucoma are slowly progressing neurodegenerative disorders, we next analysed whether the synergistic rescue effect of GDNF and CNTF is long-lasting. Analyses of animals 8 months after the lesion revealed that the transplanted NSCs were still viable and stably expressed CNTF or GDNF [126]. More importantly, we found that ~35% of all RGCs were still alive in eyes co-treated with the GDNF- and the CNTF-overexpressing cell line, as opposed to only ~7% in eyes treated with either the CNTF- or GDNF-expressing cell line and less than 2% in eyes treated with a control cell line. It is worth noting that there was no significant decrease in the number of surviving RGCs between the fourth and eighth month post-lesion, indicating that the co-administration of GDNF and CNTF conferred long-lasting and possibly life-long protection against injury-induced cell death (Figure 1 and Figure 2) [126].

How the cooperative, additive or synergistic neuroprotective effects of NTF combinations are conferred to RGCs is unknown. RGCs comprise a heterogenous group of projection neurons, and recent work has shown that RGC subtypes differ considerably in their susceptibility to pathological conditions and in their ability to regenerate injured axons [4,6,7]. It is tempting to speculate that different RGC subtypes require different neuroprotective factors to survive, providing a simple explanation for the more pronounced rescue effects observed after the administration of NTF combinations as compared to the separate administrations of individual NTFs. Furthermore, a combination of different neuroprotective factors might activate different pro-survival signalling pathways through binding to their cognate receptors, thereby promoting RGC survival more effectively than individually applied factors. Finally, factor combinations might mediate enhanced, additive or synergistic pro-survival effects by boosting indirect neuroprotective activities. For instance, GDNF and CNTF both stimulate Müller cells to secrete additional neuroprotective factors known to promote RGC survival, such as BDNF, FGF-2 or LIF [176,177,178], and both proteins protect against glutamate-induced neurotoxicity by increasing glutamate uptake in retinal glial cells [179,180].

## 6. Translating Cell-Based Neuroprotective Approaches to Clinical Applications

The therapeutic potential of MSCs for the treatment of optic neuropathies is being evaluated in several clinical trials (https://clinicaltrials.gov (accessed on 14 October 2021)), but information about the outcomes is limited [reviewed in: [25,46,54,181,182]].

Results from preclinical studies demonstrate that a sustained administration of NTFs or NTF combinations through transplantations of genetically modified cells is among the strategies to effectively promote RGC survival under various pathological conditions. The so-called encapsulated cell technology (ECT) represents a methodology to translate this cell-based treatment approach to the clinic. In fact, the technology has already been used to evaluate the therapeutic potential of a cell-based intravitreal delivery of CNTF in patients presenting with diverse neurodegenerative retinal disorders, including retinitis pigmentosa, geographic atrophy or macular telangiectasia type 2 [183,184,185,186,187,188]. ECT is based on human retinal pigment epithelial (RPE) cells, which are genetically modified to secrete the cytokine, and subsequently encapsulated into semipermeable hollow fiber membranes. After implantation of the encapsulated cell devices into the patient’s vitreous cavity, the cytokine is continuously released from the encapsulated cells into the aqueous humor, from where it diffuses into the dystrophic retinas. Important aspects of this treatment strategy include the possibility of adjusting the amount of the NTF released from the encapsulated cells prior to implantation and explanting the implanted cells in case of complications. Furthermore, the cell implants have a calculated half-life of more than 4 years, as estimated based on CNTF levels in the vitreous of patients [187], indicating their use for the treatment of slowly progressing optic neuropathies. Of note, clinical trials using the ECT have been initiated to evaluate the outcome of a sustained cell-based administration of CNTF in patients with ischemic optic neuropathy (ClinicalTrials.gov Identifier: NCT01411657) or glaucoma (ClinicalTrials.gov Identifier: NCT01408472, NCT02862938 and NCT04577300), but no results have been reported until now.

## 7. Summary and Conclusions

Available preclinical data demonstrate that cell-based neuroprotective approaches represent a useful means of attenuating the loss of RGCs in animal models of optic neuropathies. The outcome of intraocular transplantations of genetically nonmodified cell types on RGC survival rates and survival times can be improved through a forced expression of NTFs and/or by employing combinatorial cell-based neuroprotective strategies. The use of genetically modified clonal cell lines not only provides the possibility of delivering defined quantities of a specific NTF to dystrophic retinas but also defined quantities of multiple NTFs in defined ratios, eventually resulting in cooperative, additive or synergistic rescue effects. As most of the data discussed above were obtained in optic nerve injury models with a rapidly progressing loss of RGCs, it will be interesting to evaluate the therapeutic outcome of cell-based combinatorial treatment strategies in animal models that more closely mimic the slowly progressing loss of ganglion cells in glaucoma, the most prevalent optic neuropathy. Future preclinical studies should focus on the long-term impact of cell-based neuroprotective approaches on both ganglion cell survival and ganglion cell function to eventually develop treatment options for optic neuropathies for which there are currently no effective therapies available.

## Figures and Tables

**Table 1:** Promotion of RGC survival with genetically modified cells in vitro and in vivo.

Model	Cell Type	Neuroprotective Factor	Modification of Cells; Route of Cell Injection	Key Findings	Reference
RGC culture	ASs	BDNF	- retroviral transduction - cultivation of dissociated embryonic rat retinas in conditioned medium from BDNF-overexpressing ASs	significantly enhanced survival of RGCs compared to conditioned medium from control ASs	[119]
RGC-5 culture	MSCs	BDNF	- lentiviral transduction - co-culture of the RGC-5 cell line with BDNF-overexpressing MSCs	significantly enhanced survival of glutamate- or H_2_O_2_-treated RGC-5 cells compared to control MSCs	[120]
rat; OHT	MSCs	BDNF	- lentiviral transduction - intravitreal transplantation 2 days after induction of elevated IOP	significantly enhanced survival of RGCs and improved retinal function, as assessed by ERG recordings and pupillary light reflex responses ~40 days after treatment, compared to control MSCs	[121]
rat; ONC	NPCs	BDNF	- retroviral transduction - intravitreal or subretinal cell transplantation at the time of ONC	significantly enhanced survival of RGCs 30 days after cell transplantation compared to control NPCs	[122]
rat; ONT	SCs	CNTF	- lentiviral transduction - peripheral nerve sheaths repopulated with CNTF-overexpressing SCs grafted onto lesioned optic nerves at the time of ONT	significantly enhanced RGC survival and axon regeneration 4 weeks after grafting compared to nerve sheaths repopulated with control SCs	[123]
mouse; ONC	NSCs	CNTF	- lentiviral transduction - intravitreal cell transplantation 1 day after ONC	- significantly enhanced RGC survival 8 months after cell transplantation compared to control NSCs - long-distance axon regeneration	[124,125,126]
mouse; ONC	NSCs	GDNF	- lentiviral transduction - intravitreal cell transplantation 1 day after ONC	significantly enhanced RGC survival 8 months after cell transplantation compared to control NSCs	[125,126]
mouse; OHT	NPCs	IGF-1	- lipofection - intravitreal cell transplantation 1 day after induction of elevated IOP	complete protection against IOP-induced RGC loss 30 days after cell transplantation compared to control NPCs	[127]
rat; ONT	AECs	FGF-2	- lentiviral transduction - cell transplantation into the site of injury at the time of ONT	significantly enhanced RGC survival 4 weeks after cell transplantation compared to control AECs	[128]
rat; ONC	NSCs	PEDF	- lentiviral transduction - subretinal cell transplantation at the time of ONC	- significantly enhanced RGC survival 4 weeks after cell transplantation compared to repeated injections of recombinant PEDF - stimulation of axon regrowth 4 weeks after cell transplantation compared to repeated injections of recombinant PEDF	[129]
rat; ONC	NPCs	CRYBB2	- nucleofection - intravitreal cell transplantation at the time of ONC	significantly enhanced RGC survival 4 weeks after cell transplantation compared to vehicle injections	[130]

AECs: amniotic epithelial cells; ASs: astrocytes; BDNF: brain-derived neurotrophic factor; CNTF: ciliary neurotrophic factor; CRYBB2: crystallin beta B2; ERG: electroretinogram; FGF-2: fibroblast growth factor-2; GDNF: glial cell line-derived neurotrophic factor; IGF-1: insulin-like growth factor-1; IOP: intraocular pressure; MSCs: mesenchymal stem cells; NPCs: neural progenitor cells; NSCs: neural stem cells; OHT: ocular hypertension; ONC: optic nerve crush; ONT: optic nerve transection; PEDF: pigment epithelium-derived factor; RGC: retinal ganglion cell; RGC-5: retinal ganglion cell line 5; SCs: Schwann cells.

**Table 2:** Boosting RGC survival with combinatorial neuroprotective approaches.

Model	Cell Type (s)	Treatment	Key Findings	Reference
rat; ONC	MSCs, SCs	intravitreal co-transplantations of MSCs and SCs shortly after nerve injury	significantly enhanced RGC survival 2 weeks post-lesion after co-transplantations of both cell types compared to separate transplantations of each cell type	[165]
rat; ONC	MSCs	intravitreal transplantations of MSCs at the time of nerve injury combined with intravitreal injections of a PEDF-encoding AAV vector 4 weeks prior to nerve lesion	- significantly enhanced RGC survival 4 weeks post-lesion after combined gene and cell treatment compared to cell treatment only - more axon regrowth close to the lesion site after combined gene and cell treatment compared to cell treatment only	[166]
rat; incomplete ONC	OECs	transplantations of OECs into nerve injury site combined with intravitreal injections of recombinant GDNF at the time of nerve lesion	- faster and more pronounced recovery of latency and amplitude of flash visual evoked potentials until 8 weeks after injury after the combined treatment compared to each treatment alone - more axon regrowth after the combined treatment compared to each treatment alone, as indicated by qualitative analyses	[169]
rat; ONT	OECs	transplantations of OECs at the site of nerve injury combined with intravitreal injections of recombinant CNTF at the time of nerve lesion	- significantly enhanced RGC survival 4 weeks post-lesion after the combined treatment compared to each treatment alone - more regrowing axons after the combined treatment compared to each treatment alone	[170]
rat; ONC	OECs	transplantations of OECs and olfactory nerve FBs at the site of nerve injury combined with intravitreal injections of recombinant α-crystallin at the time of nerve lesion	- significantly enhanced RGC survival 4 weeks post-lesion after the combined treatment compared to each treatment alone - longer regrowing axons after the combined treatment compared to each treatment alone until 1 month post-lesion.	[171]
rat; OHT	RSCs	intravitreal transplantations of RSCs 7 days after induction of an elevated IOP and vaccination with copolymer-1	significantly enhanced RGC survival 3 weeks after elevated IOP induction after the combined treatment compared to each treatment alone	[172]
rat; ONT	FBs	intravitreal co-transplantations of FGF-2, NT-3 or BDNF-overexpressing FBs at the time of nerve lesion	significantly more surviving RGCs with axons extending at least 2 mm into the distal nerve stump 20 days after the combined treatment compared to cell-based treatments with FGF-2 and NT-3, FGF-2 and BDNF, or each individual factor	[173]
mouse; ONC	NSCs	intravitreal co-transplantations of CNTF- and GDNF-overexpressing clonal NSC lines 1 day after nerve lesion	- significantly enhanced RGC survival 8 months post-lesion after the combined treatment compared to separate transplantations of each cell line - no improved axon regeneration 1 month post-lesion after the combined treatment compared to retinas treated with CNTF only	[125,126]

AAV: adeno-associated virus; BDNF: brain-derived neurotrophic factor; CNTF: ciliary neurotrophic factor; FBs: fibroblasts; FGF-2: fibroblast growth factor-2; GDNF: glial cell line-derived neurotrophic factor; IOP: intraocular pressure; MSCs: mesenchymal stem cells; NSCs: neural stem cells; NT-3: neurotrophin-3; OECs: olfactory ensheathing cells; OHT: ocular hypertension; ONC: optic nerve crush; ONT: optic nerve transection; PEDF: pigment epithelium-derived factor; RGC: retinal ganglion cell; RSCs: retinal stem cells; SCs: Schwann cells.

## Data Availability

Not applicable.
